# Chordoma cells possess bone-dissolving activity at the bone invasion front

**DOI:** 10.1007/s13402-024-00946-6

**Published:** 2024-04-23

**Authors:** Katsuhiro Kawaai, Yumiko Oishi, Yukiko Kuroda, Ryota Tamura, Masahiro Toda, Koichi Matsuo

**Affiliations:** 1 Laboratory of Cell and Tissue Biology, Keio University School of Medicine, 35 Shinanomachi, Shinjuku-ku, 160-8582 Tokyo, Japan; 2 Department of Neurosurgery, Keio University School of Medicine, 35 Shinanomachi, Shinjuku-ku 160-8582 Tokyo, Japan

**Keywords:** Chordoma, Brachyury, Osteoclast, Bone destruction, TRAP, RANKL

## Abstract

**Purpose:**

Chordomas are malignant tumors that destroy bones, compress surrounding nerve tissues and exhibit phenotypes that recapitulate notochordal differentiation in the axial skeleton. Chordomas recur frequently, as they resist radio-chemotherapy and are difficult to completely resect, leading to repeated bone destruction and local expansion via unknown mechanisms. Here, using chordoma specimens and JHC7 chordoma cells, we asked whether chordoma cells possess bone-dissolving activity.

**Methods:**

CT imaging and histological analysis were performed to evaluate the structure and mineral density of chordoma-invaded bone and osteolytic marker expression. JHC7 cells were subjected to immunocytochemistry, imaging of cell fusion, calcium dynamics and acidic vacuoles, and bone lysis assays.

**Results:**

In patients, we found that the skull base invaded by chordoma was highly porous, showed low mineral density and contained brachyury-positive chordoma cells and conventional osteoclasts both expressing the osteolytic markers tartrate-resistant acid phosphatase (TRAP) and collagenases. JHC7 cells expressed TRAP and cathepsin K, became multinucleated via cell-cell fusion, showed spontaneous calcium oscillation, and were partly responsive to the osteoclastogenic cytokine RANKL. JHC7 cells exhibited large acidic vacuoles, and nonregulatory bone degradation without forming actin rings. Finally, bone-derived factors, calcium ions, TGF-β1, and IGF-1 enhanced JHC7 cell proliferation.

**Conclusion:**

In chordoma, we propose that in addition to conventional bone resorption by osteoclasts, chordoma cells possess bone-dissolving activity at the tumor-bone boundary. Furthermore, bone destruction and tumor expansion may occur in a positive feedback loop.

**Supplementary Information:**

The online version contains supplementary material available at 10.1007/s13402-024-00946-6.

## Introduction

Chordoma is a rare (one in a million per year) [1, 2], slow-growing malignant tumor with a phenotype that recapitulates notochord differentiation [3]. For example, chordoma cells express brachyury (T), a notochordal T-box transcription factor (TBXT) [4, 5]. Typically, chordoma occurs at the midline of the axial skeleton at the base of the skull in the clivus (35% of cases), along mobile vertebrae (15% of cases), or in the sacrococcygeal area (50% of cases), destroying axial skeleton and expanding locally. Extra-axial chordoma marked by brachyury expression has also been reported [6]. Conventional chordoma exhibits foamy, vacuolated physaliphorous cells, which are absent in poorly differentiated chordoma, which is a molecularly distinct chordoma subtype [3, 7]. A third type of chordoma is dedifferentiated chordoma, which has a biphasic appearance marked by both conventional chordoma and high-grade sarcoma [8, 9].

In skull-base chordoma emerging from the clivus, major symptoms are abnormal eye movement and swallowing disorders caused by bone destruction and compression of surrounding structures, such as the cavernous sinus and nasopharynx. Complete resection of skull-base chordoma is difficult to achieve due to proximity to the brainstem, cranial nerves, and arteries around the clivus; thus, recurrence rate after surgery is high. Radio-chemotherapy has limited effects against this tumor; thus, new therapeutic approaches such as anti-angiogenic therapy, among others, are required [10, 11]. Currently, osteolytic tumors in general are thought to recruit osteoclasts to the bone invasion front, and osteoclasts supply proteases and acids [12]. However, there is some evidence of direct bone degradation by cancer cells such as osteosarcoma [13]. It was also reported that chordoma cells express resorption markers (cathepsins, and metalloproteases) [14–16], which may indicate chordoma cells directly contribute to bone destruction. Here, we focused on cellular mechanisms driving bone destruction by chordoma.

Osteoclast cells differentiate from hematopoietic cells in the myeloid lineage and acquire bone-resorbing capacity in response to the osteoclastogenic cytokines macrophage colony-stimulating factor (M-CSF) and RANKL [17, 18]. RANKL produced by osteoblast-lineage cells binds to the receptor RANK expressed on osteoclasts and signals to effectors, such as AKT and MAP kinases, which activate transcription factors essential for osteoclastogenesis [19–21]. Mature osteoclasts resorb bone, including organic and inorganic components. To digest type I collagen (∼ 90%) and other organic bone matrix components (∼ 10%), osteoclasts secrete lysosomal cysteine proteases known as cathepsins and metalloproteinases [22, 23]. To dissolve inorganic hydroxyapatite crystals, osteoclasts secrete hydrochloric acid [24]. During osteoclastic differentiation, osteoclasts express TRAP, which dephosphorylates bone matrix proteins [25], and generate reactive oxygen species to fragmentate bone resorption products [26]. Osteoclasts also fuse to form multinucleated giant cells, cover the bone surface via an actin ring (sealing zone) to enable resorption, and display repeated oscillations in intracellular calcium levels [27, 28].

The chordoma line JHC7 (Johns Hopkins Chordoma Line 7) cells, which constitutively expresses brachyury, was established from a primary sacral chordoma sample [29]. For analysis herein, we chose JHC7 cells because these cells are highly physaliphorous (having bubbly or vacuolated appearance) observed in chordoma [30]. JHC7 cells have been successfully used to detect PD-L1 induction by pro-inflammatory cytokines [31], develop an orthotopic human chordoma model in rats [32], and analyze long non-coding RNA function [33]. Here, we analyzed chordoma specimens from patients and detected osteolytic markers in brachyury-positive clival chordoma, and further confirmed osteolytic activities in JHC7 cells. Our findings suggest that chordoma cells directly secrete proteases and acid to destroy bone.

## Results

### Tissue mineral density and porous structure of chordoma-invaded bone

**Fig. 1:**
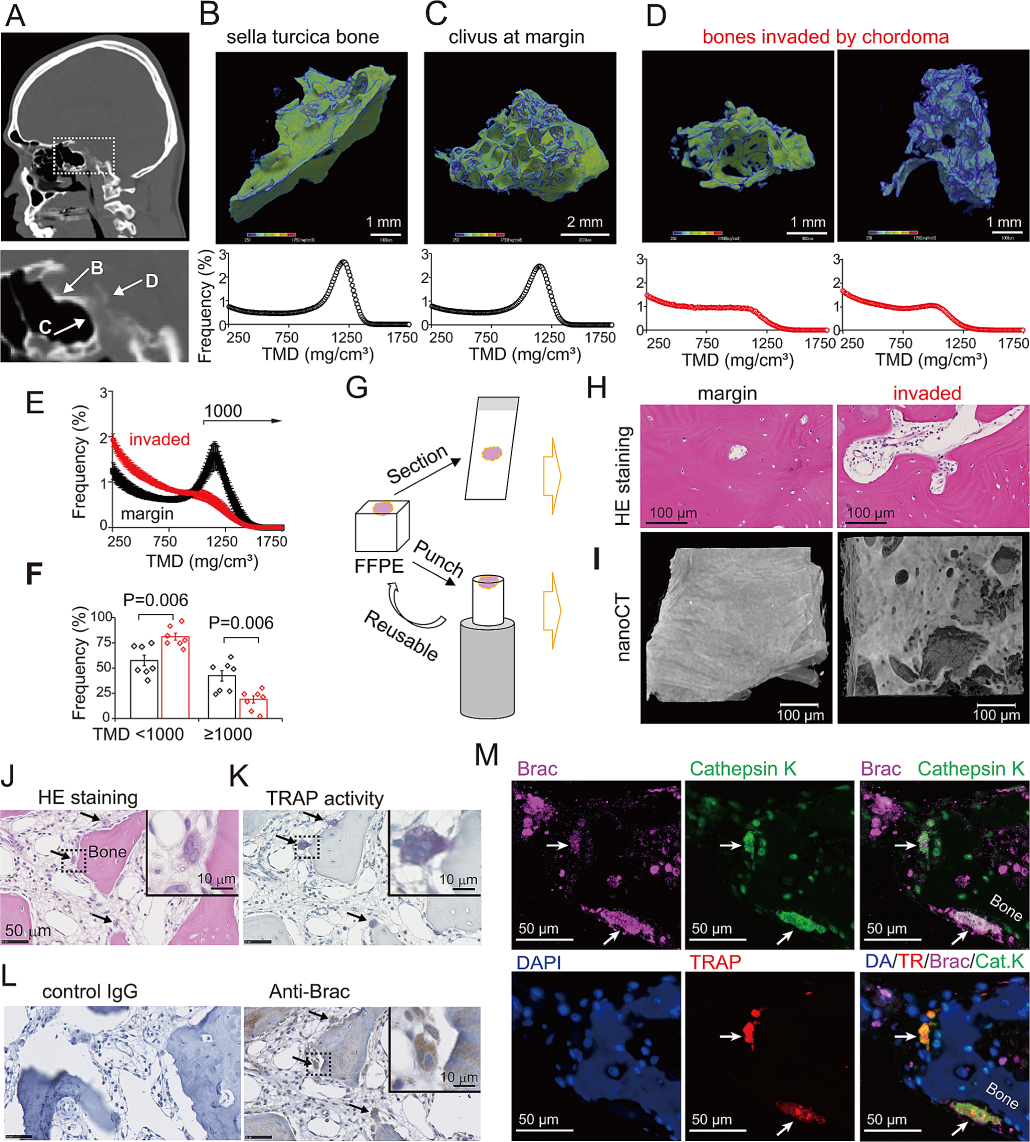
Structure and mineral density of chordoma-invaded bone and chordoma cell expression of osteolytic markers. (**A**) Upper, clinical CT sagittal image of skull of a chordoma patient. Lower, higher magnification image of dotted white box shown above. Locations of specimens shown in B, C and D are indicated by arrows. (B–D) Upper, micro-CT images (10 μm/voxel) of bone fragments derived from patients. Tissue mineral density (TMD; 250–1750 mg/cm^3^) is color-coded. Lower, frequency distributions showing TMD for each bone fragment. Shown is percentage of voxels above 250 mg/cm^3^. (**B**) Sella turcica specimen derived from a patient during chordoma surgery. (**C**) Clivus specimen at the tumor margin. (**D**) Bone tissue invaded by chordoma. (**E**) Averaged frequency distributions of TMD of specimen isolated from tissues either at tumor margins (black) or invaded by chordoma (red). Shown is percentage of voxels above 250 mg/cm^3^. Graph shows mean ± S.E.M. *N* = 7. (**F**) Quantification of data shown in graph (**E**) after separation of values below or above 1000 mg/cm^3^. Each data was plotted (black, tumor margins. red, invaded by chordoma). Mean ± S.E.M. *N* = 7. Student’s t-test. (**G**) Schema describing 2D histological analysis or 3D X-ray imaging of a FFPE sample. (**H**) H&E staining of bone tissues from chordoma specimen. Scale bar, 100 μm. (**I**) Nano-CT images (1.26 μm/voxel) of FFPE blocks. (**J**) H&E staining of a chordoma tissue section. Arrows, multinucleated giant cells. (**K**) TRAP activity staining (purple) of section neighboring the one shown in (**J**). Arrows, TRAP-positive multinuclear giant cells. (**L**) Immunostaining for control IgG (left) or brachyury (Brac; brown, right) in a chordoma tissue section. Arrows, Brac-positive multinuclear giant chordoma cells. (**M**) Fluorescent immunostaining of chordoma tissue. Arrows indicate cells co-expressing Brac, cathepsin K, and TRAP

To define mechanisms underlying bone destruction by chordoma, we analyzed chordoma samples from clival bones and bone of the sella turcica resected from patients undergoing surgery for chordoma (Fig. 1A). We then quantified tissue mineral density (TMD) of chordoma-invaded bones using micro-computed tomography (CT) at a voxel resolution of 10 μm. Sella turcica bone showed a high TMD, peaking around 1150 mg/cm^3^ (Fig. 1B). In chordoma samples, clivus at the margin of resected chordoma retained a trabecular structure and exhibited a high TMD peak (Fig. 1C). By contrast, clival bone massively invaded by chordoma cells showed unstructured morphology (Fig. 1D) and did not exhibit the TMD peak seen in bone at the margin (Fig. 1E). We next compared voxel frequency above 1000 mg/cm^3^ between bones at the tumor margin with those invaded by chordoma. As shown in Fig. 1F, clival bone invaded by chordoma contained a smaller number of higher TMD voxels than did clivus at the margin.

We next performed 2D and 3D histological analysis of clival bone at tumor margins or invaded by chordoma using conventional sectioning and 3D imaging by nano-CT at a resolution of 1.26 μm per voxel (Fig. 1G). H&E staining of bone tissue invaded by chordoma revealed an abundance of large holes (> 100 μm) containing typical chordoma cells (Fig. 1H, Fig. S1A). Consistent with H&E staining, the nano-CT image of bone invaded by chordoma showed porous structures (> 100 μm). By contrast, clivus at tumor margins was composed of very dense bone (Fig. 1I; Supplementary material, Movies S1 and S2). These morphological data prompted us to determine whether chordoma cells directly destroy bone.

### Chordoma cells expressing osteolytic markers at bone invasion fronts

To assess whether human chordoma cells display osteolytic features, we evaluated clinical clival chordoma tissues including bone for the presence of the osteolytic markers TRAP and cathepsin K. In those tissues we observed multinucleated giant cells (Fig. 1J) positive for TRAP activity (Fig. 1K) and brachyury expression (Fig. 1L). We then used immunofluorescence to ask whether brachyury- and TRAP-positive giant cells express cathepsin K. That analysis revealed colocalization of cathepsin K staining with brachyury and TRAP in chordoma cells (Fig. 1M). When we counted brachyury-positive and -negative TRAP-positive cells in these areas, we detected both TRAP-positive chordoma cells and conventional osteoclasts (52.6% brachyury-positive and 47.4% -negative) (Fig. S1B-D). In other words, approximately half of TRAP-positive cells were brachyury-positive chordoma cells at the bone invasion fronts.

### TRAP activity in chordoma cells responsive to RANKL

**Fig. 2:**
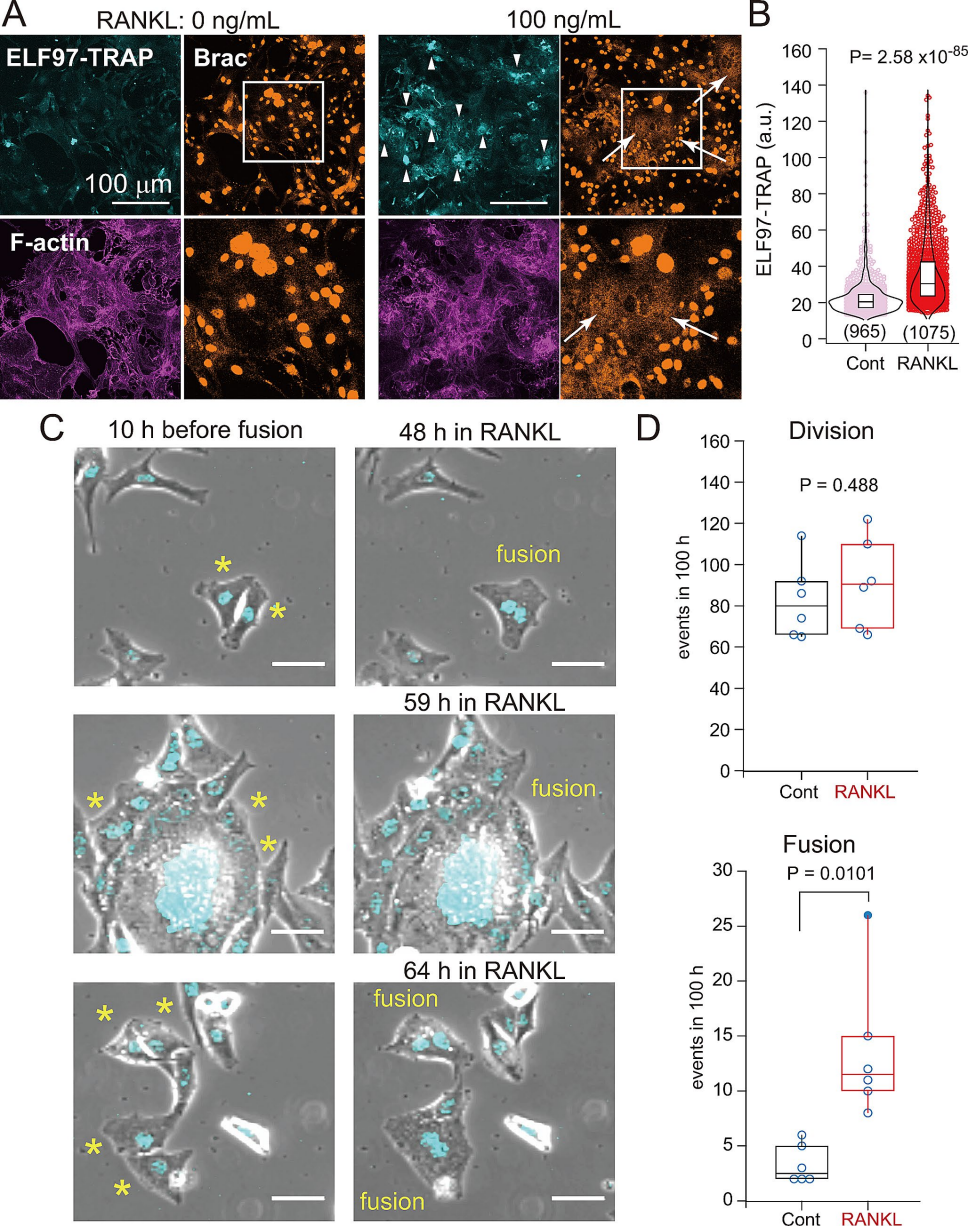
RANKL treatment increases TRAP activity and the number of fusion events in chordoma cells. (**A**) TRAP activity staining (ELF97-TRAP), brachyury (Brac) immunostaining, and phalloidin staining for F-actin in JHC7 cells treated with RANKL. Scale bar, 100 μm. Magnified view of white box is shown below. Arrowhead, JHC7 cells showing TRAP activity. Arrow, JHC7 cells showing cytoplasmic brachyury staining. (**B**) Quantitation of TRAP activity in (**A**). Violin plots and box plots are superimposed showing distribution of TRAP activity in individual cells. Total cell numbers are indicated at the bottom of each graph. Dunnett’s test compared to control without RANKL (Cont). a.u., arbitrary units. (**C**) Representative images acquired 10 h prior to JHC7 cell fusion in the presence of RANKL. Cell-cell fusion events were observed at various time points after RANKL addition. Scale bar, 50 μm. Asterisk, pre-fusion cells. (**D**) Quantitation of JHC7 cell division and fusion in the presence or absence of RANKL. Individual data points are plotted as open circles. Filled circle, outlier, Student’s t-test, *N* = 6. Reproducibility was verified through three independent experiments

RANKL, a TNF superfamily cytokine, binds the receptor RANK expressed on osteoclast precursors and osteoclasts and induces the expression of factors required for osteolysis by osteoclasts (i.e., cathepsin K, TRAP) [21]. The presence of conventional osteoclasts at chordoma invasion fronts suggests that chordoma cells are exposed to RANKL stimuli. We therefore asked whether chordoma cells are RANKL-responsive by treating JHC7 chordoma cells with or without RANKL and analyzing TRAP activity using the fluorescent phosphatase substrate ELF97. In the presence of RANKL, JHC7 cells showed overall increased TRAP activity relative to untreated controls (Fig. 2A, arrowheads). Interestingly, brachyury staining was localized to the cytoplasm rather than the nucleus in TRAP-positive giant cells using anti-Brachyury antibodies (Fig. 2A, AF2085, goat, epitope 2-202 aa; Fig. S2, 1H9A2, mouse, epitope 257–309 aa). Both antibodies stained the cytoplasm of multinucleated large JHC7 cells. Quantification of TRAP activity using ELF97 fluorescence as an indicator revealed that RANKL treatment enhanced TRAP activity in JHC7 cells relative to untreated controls (Fig. 2B), confirming that JHC7 cells are responsive to RANKL.

### RANKL enhancement of chordoma cell-cell fusion

Since we observed TRAP-positive giant cells in the presence of RANKL, we performed time-lapse imaging of JHC7 chordoma cells fusing in the presence of RANKL. Figure 2C shows three representative fusion events, each at three different time points, to illustrate both prefusion (asterisks) and fused cells. Cell fusion occurred between both mono-nucleated cells (Fig. 2C, first and third rows) and mono/multi- and multi-nucleated cells (Fig. 2C, second row) (Supplementary material, Movie S3-5). We next quantified both fusion events in the absence or presence of RANKL (Fig. 2D) and cell division events. The number of cell fusion events increased in the presence of RANKL, while the number of cell division events was unchanged. We also analyzed the time course of fusion events in individual experiment (6 dishes each for control and RANKL) (Fig. S3A). Fusion events occurred most frequently between 50 and 60 h after RANKL addition (Fig. S3B). In the absence of RANKL, cell fusion events fluctuated at a much lower frequency. These data suggest that JHC7 chordoma cell fusion is enhanced by the presence of RANKL.

### RANK signaling and protease expression by large chordoma cells

**Fig. 3:**
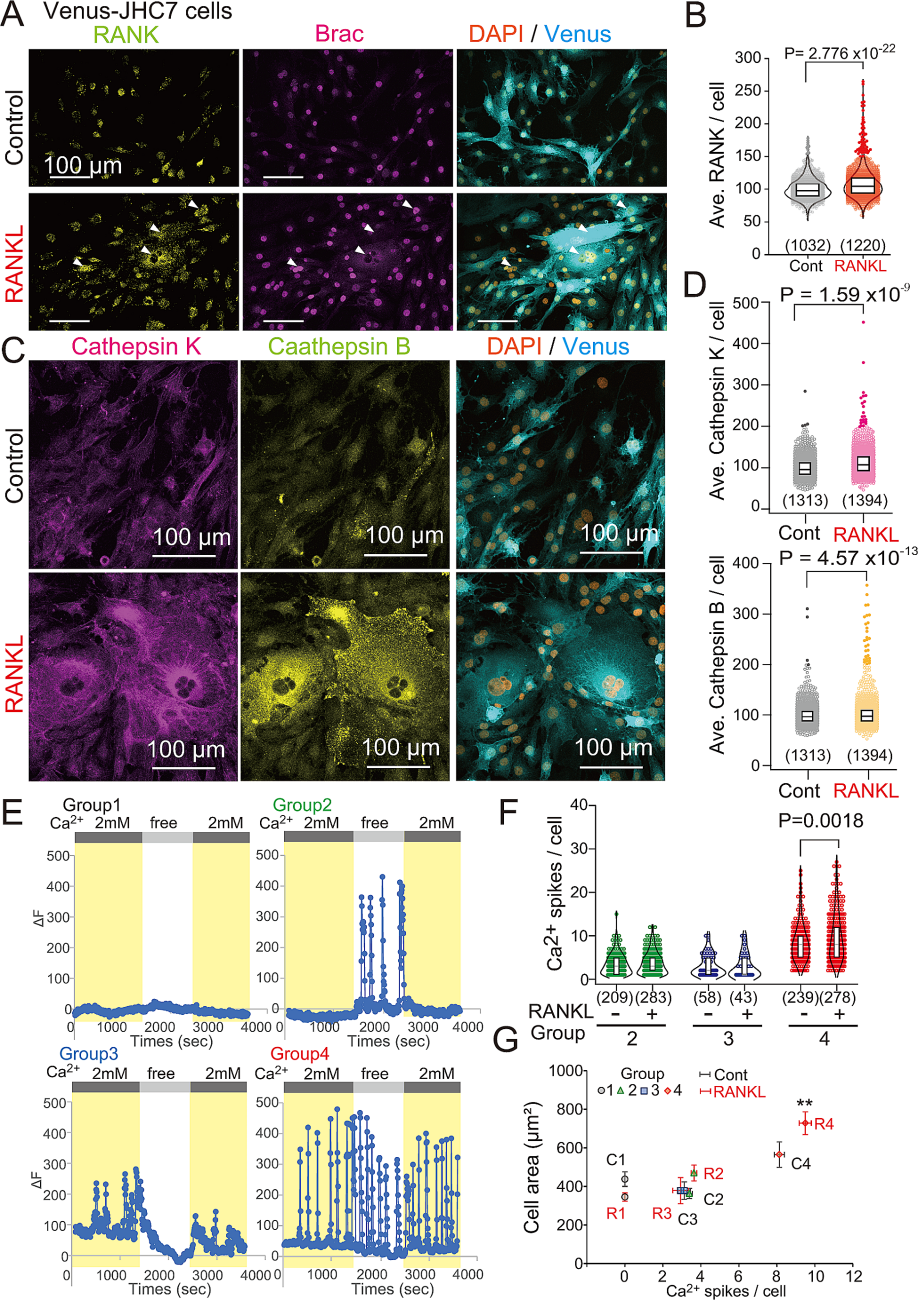
RANKL treatment enhances cathepsin K and B expression and spontaneous calcium oscillation in chordoma cells. (**A**) Immunocytochemical analysis of RANK and brachyury (Brac) expression in Venus- JHC7 cells, in the presence or absence of RANKL. Scale bar, 100 μm. (**B**) Quantitation of RANK immunosignals shown in (**A**). Data were normalized to the average intensity of the control. Total cell numbers are indicated at the bottom of each graph. Student’s t-test (95% CI, Cont: 99.0–101.0, RANKL: 106.8–109.4). (**C**) Immunocytochemical analysis of cathepsin K and B in Venus-JHC7 cells. (**D**) Quantitation of cathepsin K and B immunosignals in (**C**). Data were normalized to average intensities of controls. Total cell numbers are indicated at the bottom of each graph. Student’s t-test (95% CI, cathepsin K, Cont: 98.7–101.3, RANKL: 111.1–114.4, cathepsin B, Cont: 98.9–101.1, RANKL: 101.6–104.8). (**E**) Representative plots showing changes in intracellular calcium in RANKL-treated JHC7 cells. Groups 1–4, four spike patterns observed in response to changes in extracellular calcium concentration. ΔF, Fluo-4 fluorescence differences calculated after subtracting average baseline intensity (determined during the last 1 min in nominally calcium-free buffer). (**F**) Violin plots showing total number of calcium spikes in each cell. Total cell numbers are indicated in each graph. Student’s t-test (Group 4, 95% CI, RANKL[-]: 8.15–8.22, RANKL[+]: 9.46–9.53). (**G**) Scatter plot showing relationship between cell area and calcium spikes in each group (C1-4, Cont group 1–4, R1-4, RANKL group 1–4). Average cell area and average calcium spikes were plotted. Mean ± S.E.M. ** *P* < 0.01, Dunnett’s test compared to R4, *P* values (R1, 4.05 × 10^− 6^. R2, 4.97 × 10^− 3^. R3, 4.54 × 10^− 5^)

To visualize cell areas of JHC7 cells with heterogeneous morphology, we first stably transformed JHC7 cells with the Venus fluorescent marker [34] (Fig. 3A) and then performed RNAK immunocytochemistry. JHC7 cells expressed RANK in the absence of RANKL, but RANK expression was significantly enhanced by RANKL treatment (Fig. 3B). Given that RANK signaling leads to AKT phosphorylation in osteoclasts, we assayed ratios of phosphor-AKT to total AKT in the presence or absence of RANKL in Venus-JHC7 cells and observed significantly increased AKT phosphorylation in RANKL-treated cells (Fig. S4A, B). Osteoclasts produce proteases, such as cathepsin K, during RANKL-stimulated differentiation. Using immunocytochemistry of RANKL-treated and -untreated Venus-JHC7 cells, we calculated the average cathepsin K and cathepsin B expression per cell and observed that RANKL treatment enhanced expression of both (Fig. 3C, D). Scatter plots of areas of individual cells combined with analysis of cathepsin K expression revealed emergence of larger JHC7 cells with relatively higher cathepsin K expression after RANKL stimulation (Fig. S5A). Analysis of cathepsin B expression revealed comparable effects (Fig. S5B). These data suggest that RANKL upregulates production of collagenolytic enzymes in large chordoma cells.

### Oscillatory calcium spikes in chordoma cells increased by RANKL treatment

RANKL signaling induces spontaneous calcium oscillation in osteoclasts [28]. We therefore asked whether JHC7 chordoma cells exhibit comparable changes in intracellular calcium in the presence or absence of extracellular calcium ions, using the calcium indicator Fluo-4. Interestingly, most JHC7 cells displayed 4 patterns of spontaneous calcium oscillation (Movie S6): Group 1 cells showed no calcium oscillation, irrespective of extracellular calcium concentration (Fig. 3E, Group 1), Group 2 showed calcium oscillation only in a calcium-free environment but not in the presence of extracellular calcium (Fig. 3E, Group 2), Group 3 exhibited calcium oscillation only in the presence of extracellular calcium (Fig. 3E, Group 3), and Group 4 displayed calcium oscillation in the presence or absence of extracellular calcium (Fig. 3E, Group 4). These data indicate that JHC7 cells are heterogeneous and harbor distinct responsiveness to extracellular calcium.

We next measured the number of calcium spikes in single JHC7 cells from all 4 groups. In Group 4, the total number of Ca^2+^ spikes per cell was significantly higher in RANKL-treated versus -untreated cells (Fig. 3F). When we compared cell areas in each group, Group 4 cells were larger than those in other groups, particularly in the presence of RANKL (Fig. 3G, Fig. S5C). These data suggest that RANKL treatment enhances calcium oscillation in a subpopulation of chordoma cells (Group 4), which displays larger cell areas than other subpopulations (Groups 1–3).

### Acidic vacuoles and dissolution of calcium apatite and bone

**Fig. 4:**
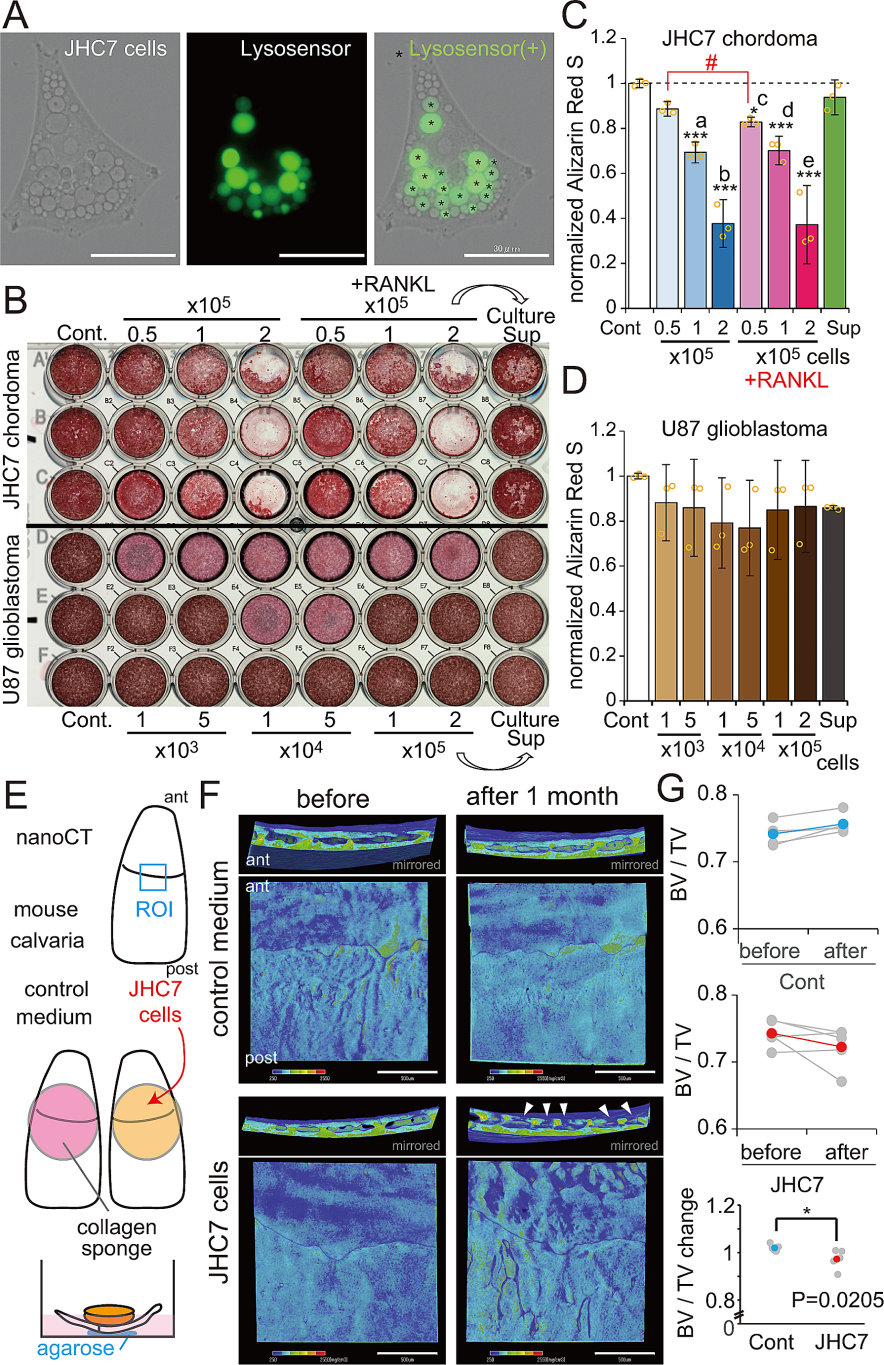
Chordoma cells exhibit acid vacuoles and lyse hydroxyapatite. (**A**) Fluorescent imaging of acid vacuoles in JHC7 cells, detected using the Lysosensor. Scale bars, 30 μm. (**B**) Upper, alizarin red S-staining of osteolytic assay plates to detect residual hydroxyapatite after culturing JHC7 cells for 15 days. Numbers of cells added to respective wells are shown at the top. Cont., no cells. Culture Sup, filtered supernatant derived from medium in the adjacent left well was added repeatedly in each medium change. Lower, negative control U87 glioblastoma cells subjected to parallel analysis. (**C**, **D**) Quantitation of alizarin red S intensity shown in (**B**), plotted for JHC7 (**C**) and U87 (**D**) cells. Individual data are also plotted. Mean ± S.E.M. *N* = 3 for each cell type. * *P* < 0.05, *** *P* < 0.001. Dunnett’s test compared to control without cells. *P* values (a, 5.88 × 10^− 5^. b, 1.77 × 10^− 6^. c, 0.0138. d, 7.82 × 10^− 5^. e, 1.78 × 10^− 6^). # P (0.028, Student’s t-test) (**E**) Schematic illustration of JHC7 cell culture on the inner surface (brain side) of a mouse calvaria. ant, anterior, post, posterior. A region of interest (ROI) (1.5 mm x 1.5 mm) was set across frontal and parietal bones. A collagen sponge with or without JHC7 cells was placed over the ROI of calvarial bone fixed in a culture dish by agarose. (**F**) Representative nano-CT images (2.5 μm/voxel) in the ROI of calvarial bone before and after 1 month of JHC7 cell culture. Tissue mineral density (TMD) (250–2550 mg/cm^3^) is color-coded. Scale bars, 500 μm. Mirrored images of top side views with matching left and right sides are indicated above. Arrowheads, indicate holes reaching the diploic space of the cranial bone due to osteolysis by JHC7 cells. (**G**) Quantitation of bone volume shown in (**F**). Individual data are plotted as gray, and averages are indicated in blue or red. *N* = 5, * *P* < 0.05. BV, bone volume (bone: 500–2550 mg/cm^3^), TV, tissue volume (tissue: 20–2550 mg/cm^3^)

Hydrochloric acid secretion is a means to dissolve hydroxy apatite crystals. Using a pH-sensitive probe (Lysosensor), we were able to visualize acidic vacuoles in JHC7 cells (Fig. 4A). Interestingly, such physaliphorous cells showed multiple acidic vacuoles in the cytoplasm, in addition to non-acidic vacuoles. We then asked whether JHC7 cells can release hydroxyapatite by growing JHC7 cells on bone resorption assay plates for 15 days and then staining plates with alizarin red S to assess calcium removal. That analysis indicated that JHC7 cells release calcium from the bottom of the hydroxyapatite-coated well in the absence and presence of RANKL (Fig. 4B, Fig. S6A). Note that cell-free conditioned medium (Culture Sup in Fig. 4B) refreshed at every medium change only marginally removed calcium from the plates. To consider the effect of glycolysis by tumor cells in this assay, we used U87 glioblastoma cells, which show elevated glycolysis [35] for comparable analysis. Because chordoma is slow-growing tumor and JHC7 cells show a slower doubling time (7 days) compared to rapidly growing U87 cells (1.5 days), various number of U87 cells were cultured on hydroxyapatite-coated plate. At any cell density, control U87 glioblastoma cells did not indicate calcium resorption activity. Quantitative analysis showed that calcium removal by JHC7 cells increased dependently on cell number. Curiously, calcium removal was also enhanced slightly but significantly by RANKL under low cell density condition (Fig. 4C). As expected, U87 control cells exhibited no resorption activity (Fig. 4D). We also analyzed another chordoma U-CH1 cells and confirmed that they release calcium from a hydroxyapatite plate, similar to JHC7 cells (Fig. S6B-D). Next, we asked whether JHC7 cells dissolve bone by growing JHC7 cells on mouse calvarial bone. JHC7 cells were cultured one month in a collagen sponge placed on calvarial bone decellularized in liquid nitrogen (Fig. 4E), and bone mineral density of calvarial bone was analyzed using nano-CT before and after culture (Fig. 4F). Bone volume of calvarial bone was significantly reduced in culture of JHC7 cells compared to controls (Fig. 4G), strongly suggesting that chordoma cells can solubilize calcium from bone.

### Enhanced chordoma cell proliferation in the presence of Ca^2+^, TGF-β1, or IGF-1

**Fig. 5:**
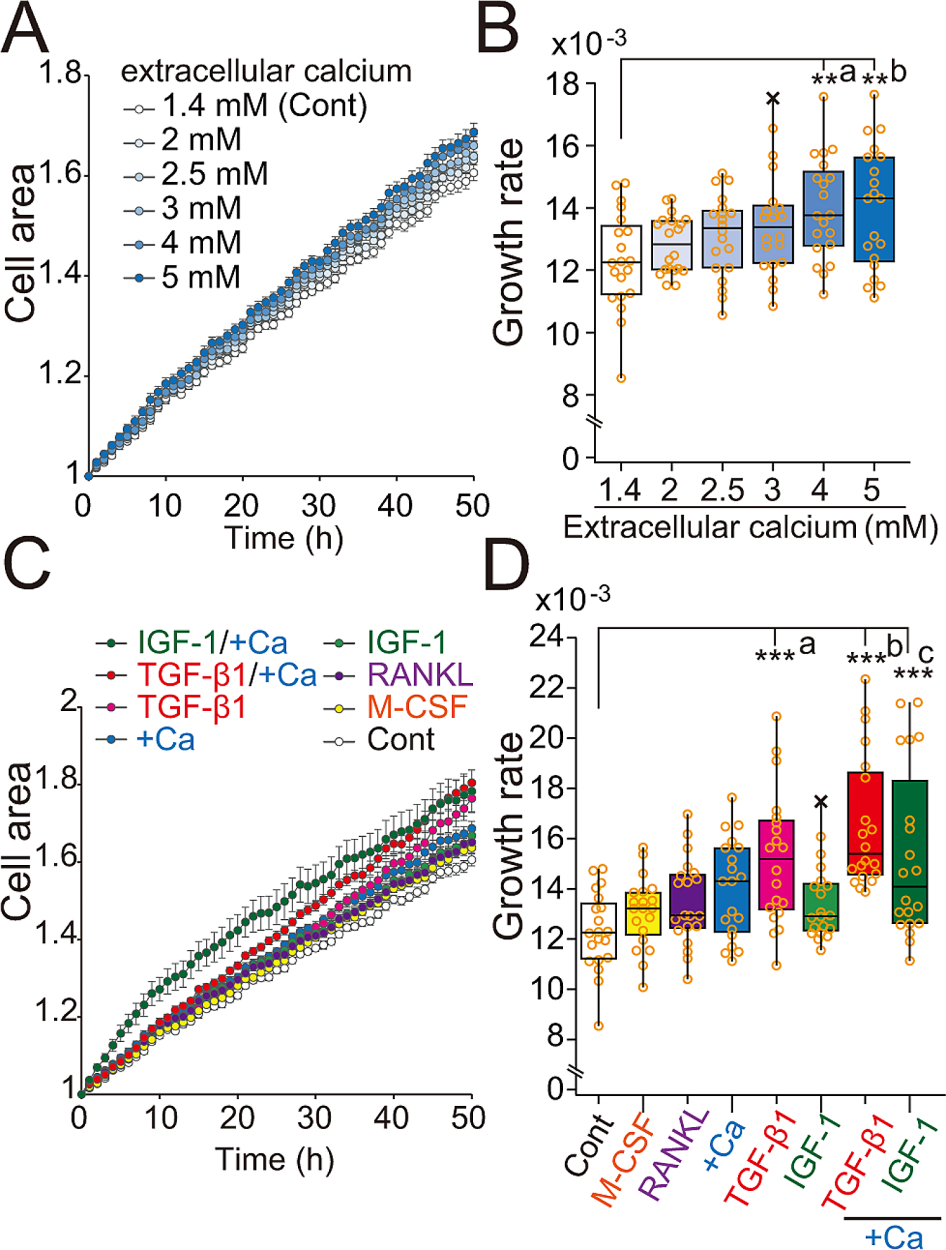
Extracellular calcium ions, TGF-β1, or IGF-1 enhance chordoma cell growth. (**A**) Growth curves of JHC7 cells cultured in different extracellular calcium concentrations. (**B**) Growth rate calculated from data shown in (**A**). Growth rates were calculated by linear regression of the slope (cell area/time, over 0–50 h). Data points are plotted as open circles. X, outlier. *N* = 20, ** *P* < 0.01, Dunnett’s test compared to control medium, *P* values (a, 0.00436, b, 0.00418). (**C**) Growth curves of JHC7 cells cultured with indicated factors. +Ca: indicates that 3.6 mM calcium was added to media already containing 1.4 mM calcium for a final concentration of 5 mM. (**D**) Quantification of data shown in (**C**). Individual data from each well are plotted as open circles. X, outlier. *N* = 20, *** *P* < 0.001, Dunnett’s test relative to control medium, *P* values (a, 0.000399, b, 0.000203, c, 1.860 × 10^− 6^)

**Fig. 6:**
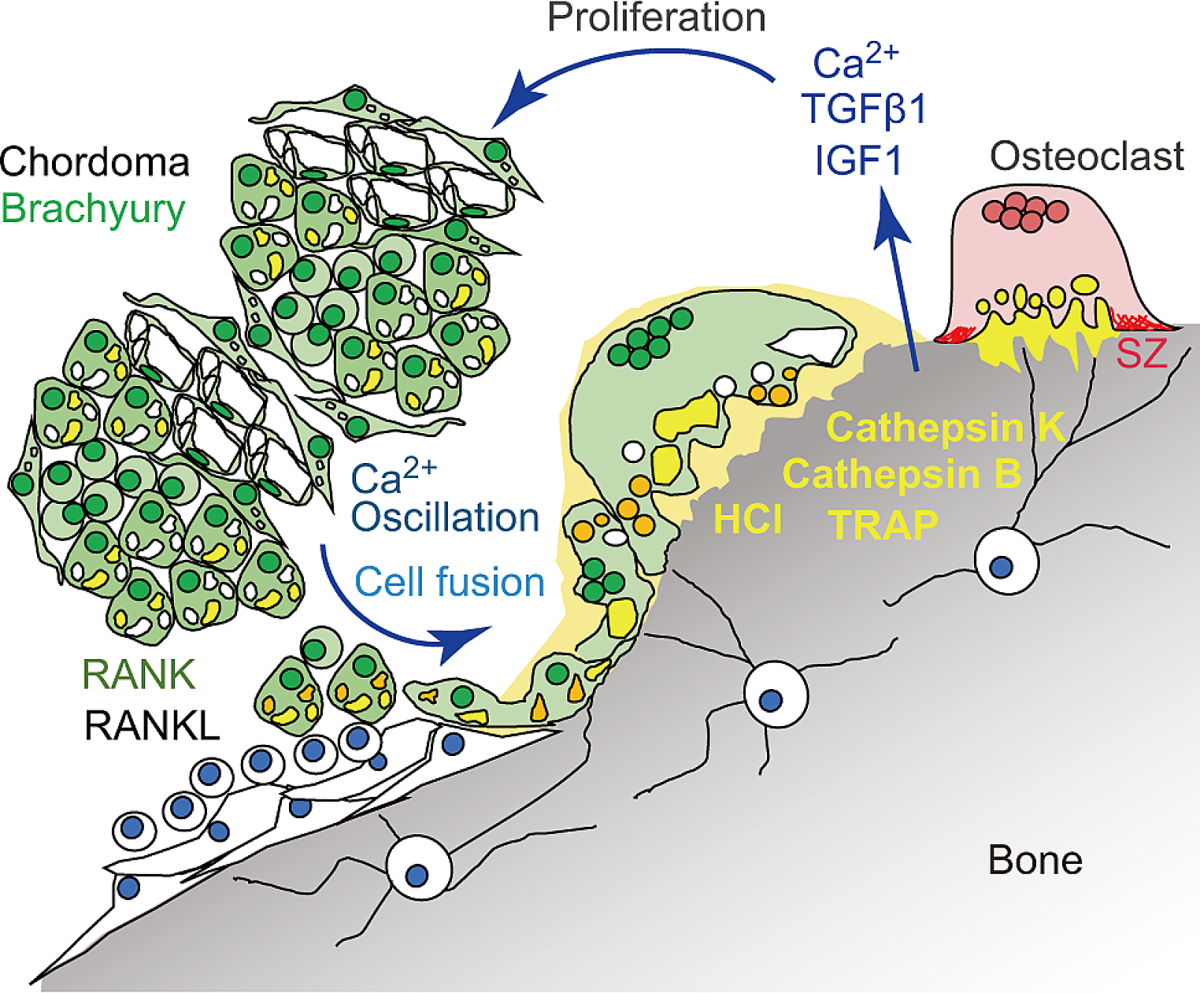
Schematic showing proposed positive feedback loop linking bone destruction with chordoma cell proliferation. Both conventional osteoclasts and an osteolytic subpopulation of chordoma cells dissolve bone and release Ca^2+^, TGF-β1 and IGF-1, which in turn enhance chordoma cell proliferation. Note that an actin ring (SZ, sealing zone) is missing from osteolytic chordoma cells

Ca^2+^ions, transforming growth factor-β1 (TGF-β1), and insulin-like growth factor-1 (IGF-1) are reportedly released from bone matrix by osteoclastic resorption [36, 37]. Therefore, we asked whether chordoma cell proliferation is altered by any of these factors. To do so, we cultured JHC7 cells in an extracellular calcium gradient and measured total cell area, as an indicator of cell growth (Fig. 5A). JHC7 cell proliferation in normal media containing 1.4 mM calcium gradually increased following addition of extracellular calcium up to 5.0 mM (final concentration), suggesting that extracellular calcium enhances proliferation (Fig. 5B). We then added M-CSF, RANKL, TGF-β1, or IGF-1 to the culture medium and assayed proliferation (Fig. 5C). M-CSF or RANKL treatment did not alter JHC7 cell growth at 1.4 mM calcium, while TGF-β1 significantly enhanced JHC7 cell growth at both 1.4- and 5.0 mM extracellular calcium (Fig. 5D). IGF-1 also significantly enhanced JHC7 cell growth in the presence of 5.0 mM extracellular calcium. These data suggest the existence of a positive feedback loop between bone destruction and chordoma cell proliferation mediated by factors released from bone (Fig. 6).

## Discussion

Here, we show that chordoma cells exhibit osteolytic features namely, acid secretion, RANK expression, and responsiveness to RANKL-leading to increased AKT phosphorylation, cathepsin K and B expression, cell fusion, and autonomous calcium oscillation. Specifically, in human chordoma specimens obtained after surgery, we observed that tissue mineral density was reduced in fragments of bone invaded by chordoma. Higher resolution analysis using histology and nano-CT revealed porous structures of 100 μm diameter or more containing chordoma cells, indicative of bone destruction. As expected, we detected brachyury-negative conventional TRAP-positive osteoclasts in invaded bone regions, suggesting that space-occupying chordoma induces indirect bone resorption by activating osteoclasts. However, we also detected brachyury-positive chordoma cells, which were also TRAP-positive, in invaded regions. Normal osteoclasts generally do not express brachyury, and if they do, express it at levels much lower than seen in chordoma cells, based on the publicly available gene expression datasets GSE37219 [38] and GSE30160. Thus, we asked whether chordoma cells possess bone-dissolving activity and found that brachyury-positive cells in human chordoma specimens were positive for cathepsin K, a collagenolytic enzyme produced by osteoclasts.

TRAP-positive chordoma cells were also responsive to the osteolytic cytokine RANKL. Compared with untreated controls, RANKL-treated JHC7 cells showed increased TRAP activity, as detected using the fluorescent TRAP substrate ELF97. In the presence of RANKL, JHC7 cells showed cytoplasmic brachyury localization, especially in multinucleated giant cells (Fig. 2). Brachyury is a transcription factor, and its exclusion from the nucleus likely reduces expression of target genes. Interestingly, osteoprotegerin (OPG), a decoy RANKL receptor [39, 40], is a known brachyury target [41, 42]. Thus, OPG down-regulation due to brachyury translocation to the cytoplasm might enhance RANK activation and further induce osteolytic phenotypes in chordoma cells. At present, mechanisms underlying changes in brachyury subcellular localization are unknown.

Cell-cell fusion is an intriguing feature of JHC7 cells revealed here in time-lapse analysis. Osteoclasts frequently undergo fusion in the presence of RANKL and M-CSF [43]. Although JHC7 cell-cell fusion was much less frequent, its frequency was significantly increased in response to RANKL. Phosphorylation of AKT, a downstream signaling factor in the RANKL-RANK pathway, increased in JHC7 cells after RANKL stimulation, and expression of cathepsin K and B significantly increased in the presence of RANKL, suggesting that chordoma cells can degrade organic bone more efficiently in the presence of RANKL.

Another remarkable feature of JHC7 chordoma cells is spontaneous calcium oscillation, also seen in osteoclasts during differentiation, and bone resorption [27, 28]. Here, RANKL treatment increased autonomous calcium oscillation frequency in a JHC7 cell subpopulation. Because JHC7 cells show changes in intracellular calcium oscillation dependent on extracellular calcium, JHC7 cells can apparently sense extracellular calcium concentrations similarly to osteoclasts [44]. JHC7 chordoma cells also contain large acidic vacuoles and solubilize calcium from hydroxyapatite and mouse calvarial bone. These properties are also shared by osteoclasts, which contain large acidic vacuoles essential for bone resorption [45].

Overall, these data indicate that chordoma cells utilize molecular mechanisms partially overlapping with osteoclasts. On the other hand, JHC7 cells and osteoclasts exhibit key differences: while initiation of calcium oscillation and cell-cell fusion in osteoclasts depends on RANKL, a subpopulation of JHC7 cells showed spontaneous calcium oscillation and cell fusion in the absence of RANKL. And, although osteoclasts form an actin ring or sealing zone to resorb designated areas, multinucleated JHC7 cells rarely formed recognizable actin rings, based on F-acting staining (Fig. 2A), suggesting that acidification by chordoma cells is amorphous and nonregulatory, unlike osteoclast activity.

Bone destruction and tumor expansion are remarkable features of chordoma. Our analysis of JHC7 cell proliferation in the presence of extracellular calcium, TGF-β1, or IGF-1 indicates that bone destruction and tumor expansion are linked in a positive-feedback loop: we propose that growing tumors provide increased numbers of osteolytic cells that dissolve bone, releasing factors such as calcium, TGF-β1, and IGF-1 that promote chordoma cell proliferation, which might enhance bone degradation. Conventional osteoclasts recruited by chordoma likely enhance this positive feedback by releasing these factors. Consistently, TGF-β1 expression in chordoma is a reported risk factor for chordoma tumor progression [46]. TGF-β1’s contribution to tumor progression remains a matter of debated, since loss of the chromosomal locus harboring TGF-β receptors is reported in chordoma [47]. Interestingly, brachyury expression increases with the TGF-β1-induced epithelial-to-mesenchymal transition (EMT) in human cancer lines [48]. Thus, TGF-β1 activation in chordoma cells may increase brachyury expression, which is essential for their proliferation [49]. Type 1 insulin-like growth factor receptor (IGF-1R) expression has been detected in the plasma membrane and cytoplasm of chordoma cells and IGF-1R inhibition is considered a therapeutic target in chordoma [50].

One limitation of this study is its reliance on the JHC7 and U-CH1 chordoma lines, which are derived from sacral chordoma [29, 51]. For example, skull-base and sacral chordomas may exhibit distinct transcriptomes [52]. Future studies using clival chordoma cell lines may provide deeper insight into the cellular mechanisms underlying bone destruction by chordoma cells. Conversely, expanding the scope beyond clival chordoma to include observations of spinal and sacral chordomas may allow generalization of the hypothetical mechanism for direct bone destruction by chordoma cells.

Here, we show that chordoma cells harbor a subpopulation exhibiting osteolytic phenotypes. It is generally accepted that bone degradation by osteolytic tumors releases cytokines from tumor tissue that induce osteoclastic differentiation of monocyte/macrophage-lineage cells [12, 53]. However, our results suggest that in addition to conventional bone resorption by osteoclasts, a subset of tumor cells exhibit osteolytic phenotypes and can degrade surrounding bone on their own.

## Materials and methods

### Patient specimens

Forty-six primary skull-base chordoma specimens from patients who underwent surgery for chordomas at Keio University Hospital from 1985 to 2019 were examined histologically for the presence of bone. Eleven specimens (from 2000 to 2019) containing bone tissue from 7 males and 4 females, with an average patient age of 51.36 years (31–78 years, SD 16.58) were used in this study. These specimens were resected by transnasal endoscopic surgery from conventional chordoma patients, Musculoskeletal Tumor Society (MSTS) staging IA (no metastasis, clivus lesion).

Specimens were washed with PBS, quickly transferred to the laboratory and fixed in 4% (wt/vol) paraformaldehyde (PFA) overnight. For micro-CT, PFA-fixed fresh specimens (14 pieces from 4 patients) were imaged in PBS. For nano-CT imaging, cylindrical samples of 3.5 mm diameter were obtained from formalin-fixed paraffin-embedded (FFPE) blocks using a biopsy punch (BP-35 F, Kai industries, Japan) [54]. Histological sections were obtained from paraffin blocks.

### Antibodies

Used in this study were mouse anti-brachyury (1H9A2, Abcam, UK), goat anti-brachyury (AF2085, R&D systems, USA), rabbit anti-TRAP (PAA902Mu01, Cloud-Clone Corp, USA), rabbit anti-AKT (#9272, Cell Signaling Technology (CST), USA), mouse Venus anti-phospho-AKT (#4051, CST), goat anti-cathepsin K (sc-6506, Santa Cruz Biotechnology, USA), rabbit anti-cathepsin B (#31,718, CST), and rabbit anti-RANK (H-300, Santa Cruz).

### Cell culture

JHC7 cells (CRL-3267, Lot:63,327,609, RRID: CVCL_L154), U-CH1 cells (CRL-3217, Lot:70,014,931, RRID: CVCL_4988) and U-87 MG cells (HTB-14, RRID: CVCL_0022) were obtained from ATCC and cultured according to the manufacturer’s instructions, (see Supplementary materials and methods for details).

### Micro- and nano-computed tomography

Micro-CT images were obtained as described [55], (see Supplementary materials and methods for details). Nano-CT images were obtained using an X-ray microscope (nano3DX, Rigaku Corporation, Japan) operated at 40 kV, 30 mA (see Supplementary materials and methods for details). During transnasal endoscopy, several pieces of tissue samples were obtained. The bone pieces that contained trabeculae were designated as “bone at margin”, while the chordoma samples containing bone pieces lacking trabeculae were designated as “bone invaded”.

### Histological analysis

Histopathological analyses were performed as described [55] (see Supplementary materials and methods for details).

### Immunostaining of chordoma cells

JHC7 cells were cultured on glass coverslips (Matsunami Glass, Japan) coated with 0.1% gelatin (G1393, Sigma-Aldrich). After 24 h, cells were treated with or without 10 ng/mL recombinant human (rh) M-CSF (R&D systems) and 100 ng/mL rhRANKL (Fujifilm Wako, Japan). Culture medium was changed every 3–4 days. After 10 days, cells were fixed in 4% PFA in PBS for 10 min. Immunostaining was performed as described [56] (see Supplementary materials and methods for details).

### Cell division and fusion imaging

JHC7 cells were cultured on glass bottomed dishes (Iwaki Glass, Japan). After 24 h, cells were loaded with 3.3 μM Hoechst 33,342 (23491-52-3, Nacalai) in culture medium at 37˚C for 20 min. Imaging was performed over 100 h in phenol red-free culture medium, with or without 100 ng/mL rhRANKL, using an Olympus FV10i confocal microscope (2 frames/hour).

### Intracellular calcium imaging

JHC7 cells were cultured on glass bottomed dishes, and after 24 h treated with or without 10 ng/mL rhM-CSF and 100 ng/mL rhRANKL for an additional 24 h. Intracellular calcium imaging was performed as described [56] (see Supplementary materials and methods for details).

### Imaging of acidic intracellular organelles

JHC7 cells were cultured on glass bottomed dishes and 24 h later loaded with 1 μM LysoSensor Yellow/Blue DND-160 (L7545, Thermo Fisher Scientific) for 30 min. Imaging was performed in BSS (see, Supplementary materials and methods) with 2 mM CaCl_2_ using a BZ-9000 fluorescence microscope (Keyence, Japan).

### In vitro acid secretion assay

JHC7 cells or U-CH1 cells were cultured on a bone resorption assay plate 48 (BRA-48P, PG Research, Japan). To enhance cell adhesion, human fibronectin (1 μg/cm^2^, F0895, Sigma-Aldrich) was used to coat half of the wells for U-CH1 cells culture. After 24 h, cells were treated with or without 10 ng/mL rhM-CSF and 100 ng/mL rhRANKL. Culture medium was changed every 3–4 days, and supernatants of wells that initially contained 2 × 10^5^ JHC7 cells or 4 × 10^5^ U-CH1 cells were passed through a 0.22 μm-filter and transferred to “culture sup” wells. After 15–16 days, the assay was stopped by 5 min of treatment with 5% (vol/vol) sodium hypochlorite (31518-35, Nacalai) and wells were washed with pure water (5 min, 3 times). Calcium phosphate was then stained with 0.5% alizarin red S, pH 7.4 (A5533, Sigma-Aldrich) and plates were scanned by Gelscan-2 (iMeasure, Japan).

### In vitro bone lysis assay

Mouse calvarial bone was collected from adult C57BL/6J mice (2–3 months old, Clea Japan) in accordance with the Institutional Guidelines on Animal Experimentation at Keio University. Calvarial bone was decellularized in liquid nitrogen and sterilized under UV. To align the position of bone before and after culture, a region of interest (ROI) of 1.5 mm x 1.5 mm was set in an area across frontal and parietal bones, and a collagen sponge with or without JHC7 cells was put over the ROI of calvarial bone fixed in the culture dish by agarose. Tissue mineral density of calvarial bone was analyzed using nano-CT, before and after one month of culture.

### Cell proliferation assay

JHC7 cells were cultured on 96-well plates. After 24 h, the culture medium was replaced with medium supplemented with various concentrations of calcium and/or cytokines: 10 ng/mL rhM-CSF, 100 ng/mL rhRANKL, 50 ng/mL rmTGF-β1 [7666-MB-005/CF, R&D systems] and 100 ng/mL rmIGF-1 [791-MG-050, R&D systems]. Imaging and analysis of percent cell confluence (by phase contrast) were performed using an IncuCyte S3 live cell analysis system (Sartorius, Germany). To generate growth curves, cell areas were normalized to t = 0. Growth rates were calculated by linear regression of the slope (over 0–50 h).

### Statistical analysis

Statistical comparison of two independent groups of data was performed using the Student’s t test. Other statistical analysis was conducted using SPSS Statics version 27 (IBM Corp., USA) or IgorPro 9.0 software (HULINKS Inc., Japan).

## Electronic supplementary material

Below is the link to the electronic supplementary material.

**Supplementary Material 1:** Supplementary materials and methods

**Supplementary Material 2: Fig. S1** Brachyury-positive or -negative cells expressing TRAP at the tumor-bone boundary of chordoma tissues. (A) Immunostaining for control IgG (left) or brachyury (Brac; brown, right) in bone tissue section including chordoma cells. Scale bar, 100 µm. (B) Fluorescent immunostaining of chordoma tissue. Arrows indicate conventional osteoclasts expressing TRAP. Filled arrowheads indicate cells co-expressing brachyury and TRAP. Open arrowheads indicate conventional chordoma cells expressing brachyury. Dotted line, bone tissue. Magnified views of small white boxes in each panel are inserted. Scale bars in magnified views, 20 µm. (C) Quantitation of brachyury in TRAP-expressing cells in (A). Scatter plot of TRAP and brachyury expression in individual cells. TRAP-positive cells (5 chordoma specimens including bones) totalled 154. (D) Histogram showing distribution of brachyury-positive or -negative cells. **Fig. S2** Brachyury expression in JHC7 cells. (A) Co-immunostaining of JHC7 cells using normal IgG. (B) Co-immunostaining of JHC7 cells using two anti-Brachyury antibodies, which recognize the different epitopes (AF2085: 2-202 aa, 1H9A2: 257–309 aa). Arrows, multinucleated large JHC7 cells stained with both brachyury antibodies in cytoplasm. Scale bar, 30 µm. **Fig. S3** Time course of fusion events in JHC7 cell culture. (A) Each fusion event was plotted in chronological order after RANKL addition. (B) Histogram showing distribution of total fusion events every 10 hours in (A). **Fig. S4** AKT phosphorylation in JHC7 cells. (A) Immunocytochemical analysis of levels of phospho- (red) and total (green) AKT in Venus-JHC7 cells, in the presence or absence of RANKL. Scale bar, 200 µm. (B) Quantitation of AKT phosphorylation in (A). Violin plots show distribution of AKT phosphorylation (phosphorylated AKT/total AKT). Data were normalized to the average ratio (phospho-AKT(Ser473P)/total AKT) seen in control cells. Total numbers of cells are indicated at the bottom of each graph. Student’s t-test (95% CI, Cont: 0.992–1.008, RANKL: 1.034–1.052). **Fig. S5** Large JHC7 cells (> 1 x 10^4^ µm^2^) cultured in the presence of RANKL show high cathepsin K and B expression. (A) Upper, scatter plots of individual cell areas and relative cathepsin K expression, in the presence or absence of RANKL. Lower, histogram showing distribution of cathepsin K-positive cells. Data were normalized to average intensities of controls. Dotted line indicates 150% relative to control average. (B) Upper, scatter plot of individual cell areas and relative expression of cathepsin B. Lower, histogram of cathepsin B positive cells. (C) Scatter plot showing relationship between cell area and calcium spikes in each group. Average cell area of each group (R1-4) of RANKL treated cells were indicated as bottom. Mean ± S.E.M. ** P < 0.01, Dunnett’s test compared to Group 4, P values (Group 1, 4.05 x10^-6^. Group 2, 4.97 x 10^− 3^. Group 3, 4.54 x10^-5^). **Fig. S6** U-CH1 cells lyse hydroxyapatite. (A) Magnified image of alizarin red S staining in Fig. 4B. top wells (A1-A8). (B) Upper, U-CH1 cells were cultured with and without RANKL on osteolytic assay plates coated with hydroxyapatite, and 16 days later, residual hydroxyapatite on plates was stained with alizarin red S. Numbers of cells added to respective wells are shown along the top. Cont., no cells. Sup, filtered supernatant derived from medium of the adjacent left was repeatedly added at each medium change. Lower, U-CH1 cells subjected to comparable analysis using fibronectin (FN)-coated plates. (C, D) Quantitation of alizarin red S intensity shown in (B), plotted for U-CH1 cells cultured on uncoated (C) and FN-coated (D) plates. Individual data are plotted. Mean ± S.E.M. N = 3. *** p < 0.001 vs Cont. ### p < 0.001 vs Sup. Dunnett’s test

**Supplementary Material 3: Movie S1**: Nano-CT image of FFPE block of a specimen isolated from tissues at the tumor margin

**Supplementary Material 4: Movie S2**: Nano-CT image of FFPE block of a specimen isolated from tissues invaded by chordoma

**Supplementary Material 5: Movie S3**: Representative live cell imaging of JHC7 cells. This movie shows cells shown in the first row in Fig. 2C. Nuclei were stained with Hoechst 33324 before imaging. *, prefusion cells

**Supplementary Material 6: Movie S4**: Representative live cell imaging of JHC7 cells. This movie shows cells shown in the second row in Fig. 2C. Nuclei were stained with Hoechst 33324 before imaging

**Supplementary Material 7: Movie S5**: Representative live cell imaging of JHC7 cells. This movie shows cells shown in the third row in Fig. 2C. Nuclei were stained with Hoechst 33324 before imaging. *, prefusion cells

**Supplementary Material 8: Movie S6**: Representative intracellular calcium imaging of JHC7 cells. 2 mM Ca^2+^: extracellular buffer containing 2 mM calcium; Ca^2+^ free: nominally calcium-free buffer

## Data Availability

No datasets were generated or analysed during the current study.
