# Supplementary material for: Chordoma cells possess bone-dissolving activity at the bone invasion front
Source: Cell Oncol (Dordr). 2024 Apr 23;47(5):1663–77. doi: 10.1007/s13402-024-00946-6 (PMC11466907; doi:10.1007/s13402-024-00946-6)
Supplement: Supplementary file 1 — Supplementary Material 1: Supplementary materials and methods [file 13402_2024_946_MOESM1_ESM.docx]

Cellular Oncology

Supplementary Materials and Methods for

**Chordoma cells possess bone-dissolving activity at the bone invasion front**

Katsuhiro Kawaai^1^, Yumiko Oishi^2^, Yukiko Kuroda^1^, Ryota Tamura^2^,

Masahiro Toda^2^, and Koichi Matsuo^1^

^1^Laboratory of Cell and Tissue Biology, Keio University School of Medicine, Tokyo, Japan.

^2^Department of Neurosurgery, Keio University School of Medicine, Tokyo, Japan.

*Corresponding author. Email: kmatsuo@keio.jp

**Cell culture**

JHC7 cells were obtained from ATCC (CRL-3267, Lot:63327609) and cultured in DMEM/Ham's F-12 medium (08460-95, Nacalai, Tokyo, Japan) supplemented with 10% fetal bovine serum and an antibiotic-antimycotic reagent (15240062, Thermo Fisher Scientific, MA, USA). U-CH1 cells (CRL-3217, Lot:70014931, RRID:CVCL_4988) Iscove's Modified Dulbecco's Medium (IMDM, 11506-05 Nacalai): RPMI-1640 Medium (30264-85, Nacalai) (4:1) supplemented with 10% fetal bovine serum, antibiotic-antimycotic reagent (15240062) and 1% L-glutamine (16948-04, Nacalai). U-87 MG cells were obtained from ATCC (HTB-14) and cultured in MEMα nucleosides medium (12571063, Thermo Fisher Scientific) supplemented with 10% fetal bovine serum and an antibiotic/antimycotic reagent. Venus-JHC7 (ffLuc [fusion protein consisting of Aequorea GFP mutant and firefly luciferase]-JHC7) cells were established by infection with the adenoviral vector CSII-EF-ffLuc [1]. Subsequent enrichment of Venus-expressors was performed using flow cytometry (MoFlo XDP, Beckman Coulter, CA, USA).

**Micro-computed tomography**

Micro-CT images were obtained using the X-ray micro-CT scanner R_mCT2 (Rigaku Corporation, Tokyo, Japan) operated at 90 kV, 160 μA, field of view 5 mm (voxel size, 10 μm). Tissue mineral density (TMD) was quantified based on a phantom containing hydroxyapatite disks (100, 300, and 400 mg/cm^3^) and an aluminum bar (1550 mg/cm^3^) (Ratoc System Engineering, Tokyo, Japan).

**Nano-computed tomography conditions**

X-ray source, Cu. X-ray detector, L1080. Field of view, 2.662 x 2.662 mm (voxel size, 1.26 μm/voxel). X-ray detector position, XD = 3 mm. Range of sample rotation angle, 180 degrees. Number of images, 1700. Exposure time, 25 sec.

**Histological analysis**

Histopathological analyses were performed on 4 μm sections of formalin-fixed, paraffin-embedded skull base chordoma tissue. Histological characteristics were assessed by hematoxylin and eosin (H&E) staining. TRAP activity staining was performed using an Acid Phosphatase, Leukocyte Kit (387A, Sigma-Aldrich, MO, USA). For IHC, sections were deparaffinized, rehydrated, and treated in 1% hydrogen peroxide at room temperature 15 min for DAB detection. Antigen retrieval was performed in 0.01 M citrate buffer (pH 6.0) at 37˚C for 30 min. Sections were blocked with blocking solution [1.0% (wt/vol) BSA, 5.0% (vol/vol) normal donkey serum (D9663, Sigma-Aldrich) and 10 μg/mL donkey IgG (017-000-003, Jackson ImmunoResearch Laboratories) in 0.02% Triton-X100/PBS (PBS-tr)] for 60 min at room temperature and then stained with indicated primary antibodies in 1.0% (wt/vol) BSA/PBS overnight at 4˚C. After three 15-min PBS-tr washes, HRP-conjugated secondary antibodies (anti-goat IgG, MP-7405, Vector Laboratories, CA, USA) was applied for 60 min at room temperature. After PBS-tr washing, sections were developed using an ImmPACT DAB kit (SK-4105, Vector Laboratories). Nuclear counterstaining was performed using hematoxylin. Sections were dehydrated and mounted with softmount (192-16301, FUJIFILM Wako Pure Chemical Corporation, Osaka, Japan). For fluorescent immunostaining, we used DAPI (diamidino-2-phenylindole, Sigma-Aldrich) for nuclear staining, Alexa plus 488/555/647-conjugated secondary antibodies (Thermo Fisher Scientific), a TrueVIEW Autofluorescence Quenching Kit (SP-8400, Vector laboratories) and ProLong Glass Antifade Mountant (P36980, Thermo Fisher Scientific). Sections were observed under a confocal laser scanning microscope FV3000 (Olympus, Tokyo, Japan).

**Immunostaining of chordoma cells**

JHC7 cells were cultured on a cover glass (Matsunami Glass, Osaka, Japan) coated with 0.1% gelatin (G1393, Sigma-Aldrich). After 24 hours, cells were treated with or without 10 ng/mL rhM-CSF and 100 ng/mL rhRANKL. Culture medium was changed every 3-4 days. After 10 days, cells were fixed in 4% (wt/vol) PFA in PBS for 10 min, and permeabilized 5 min with 0.1 % (wt/vol) Triton X-100/PBS. After incubation with blocking solution for 60 min at room temperature, cells were stained with indicated primary antibodies in 1.0% (wt/vol) BSA/PBS overnight at 4˚C. Following three 15-min PBS washes, Alexa plus 488/555/647-conjugated secondary antibodies (Thermo Fisher Scientific) or Alexa 647-conjugated Phalloidin (A22287, Thermo Fisher Scientific) and DAPI were applied for 60 min at room temperature. After PBS washing, cells were mounted with ProLong Glass Antifade mountant and observed under a confocal laser scanning microscope FV3000 (Olympus, Tokyo, Japan). For fluorescent TRAP activity staining, an ELF97 Endogenous Phosphatase Detection Kit (E6601, Thermo Fisher Scientific) was used. Quantification was performed using IMARIS software (Oxford Instruments, Abingdon, UK) or NIH Image J software. Cell areas were defined based on phalloidin staining or Venus expression.

**Intracellular calcium imaging**

JHC7 cells were cultured on a glass bottom dish (Iwaki Glass), and after 24 hours, treated with or without 10 ng/mL rhM-CSF and 100 ng/mL rhRANKL for 24 hours. Cells were then loaded with 4 μM Fluo-4 AM (F311, DOJINDO, Kumamoto, Japan) for 30 min. Imaging was performed in balanced salt solution (BSS, 20 mM HEPES, pH 7.4, 115 mM NaCl, 5.4 mM KCl, 1 mM MgCl_2_, and 10 mM glucose) with or without 2 mM CaCl_2_ using a FV3000 confocal laser scanning microscope (10 frames/min). Calcium spikes were defined as when the Fluo-4-intensity difference D(t) at frame t was 10-fold higher than average D (at baseline). Note that t is a frame number and increases every 6 sec.

D(t) = DF(t) - average DF(t-2, t-1, t, t+1, t+2).

**Reference**

1 C. Hara-Miyauchi, O. Tsuji, A. Hanyu, S. Okada, A. Yasuda, T. Fukano, C. Akazawa, M. Nakamura, T. Imamura, Y. Matsuzaki, H.J. Okano, A. Miyawaki and H. Okano, Biochem Biophys Res Commun 419, 188-193 (2012) doi: 10.1016/j.bbrc.2012.01.141
