# Supplementary figures and images for: Chordoma cells possess bone-dissolving activity at the bone invasion front
Source: Cell Oncol (Dordr). 2024 Apr 23;47(5):1663–77. doi: 10.1007/s13402-024-00946-6 (PMC11466907; doi:10.1007/s13402-024-00946-6)

Fig. S1

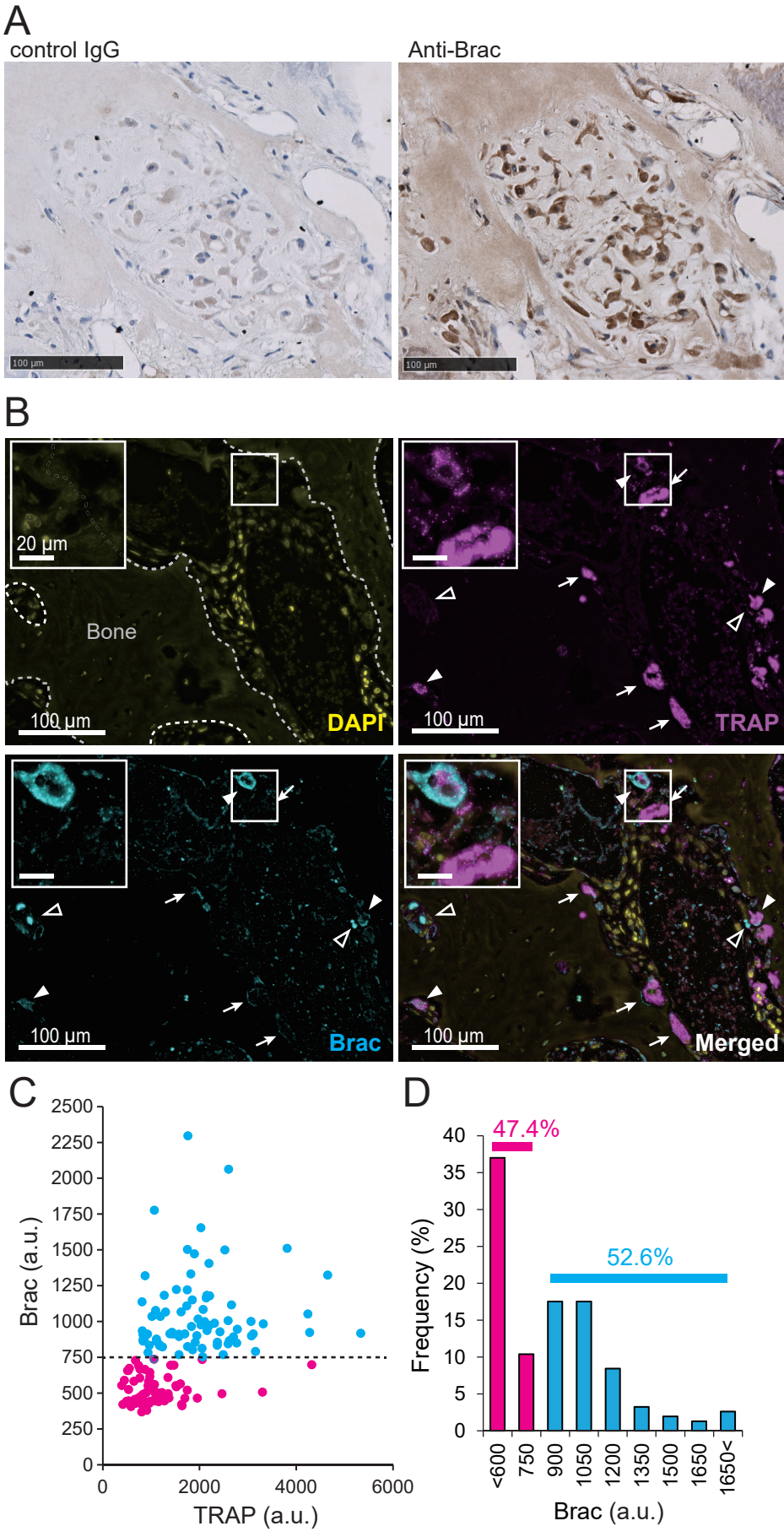

Fig. S2

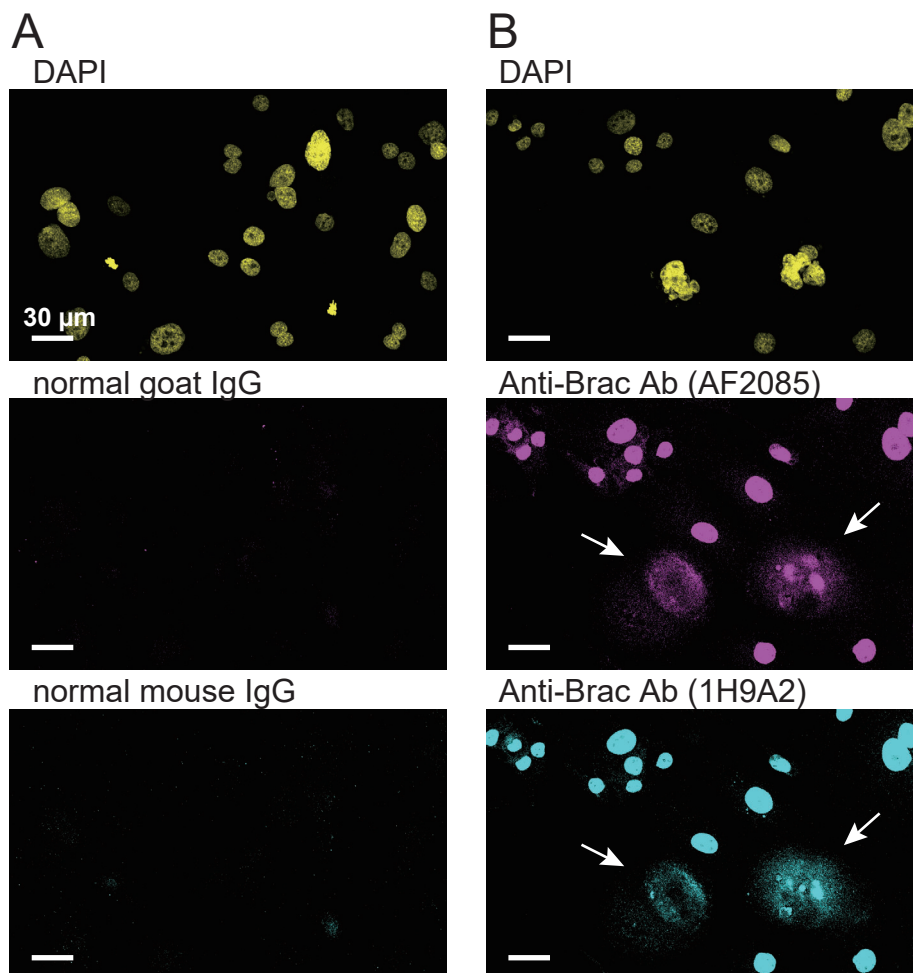

Fig. S3

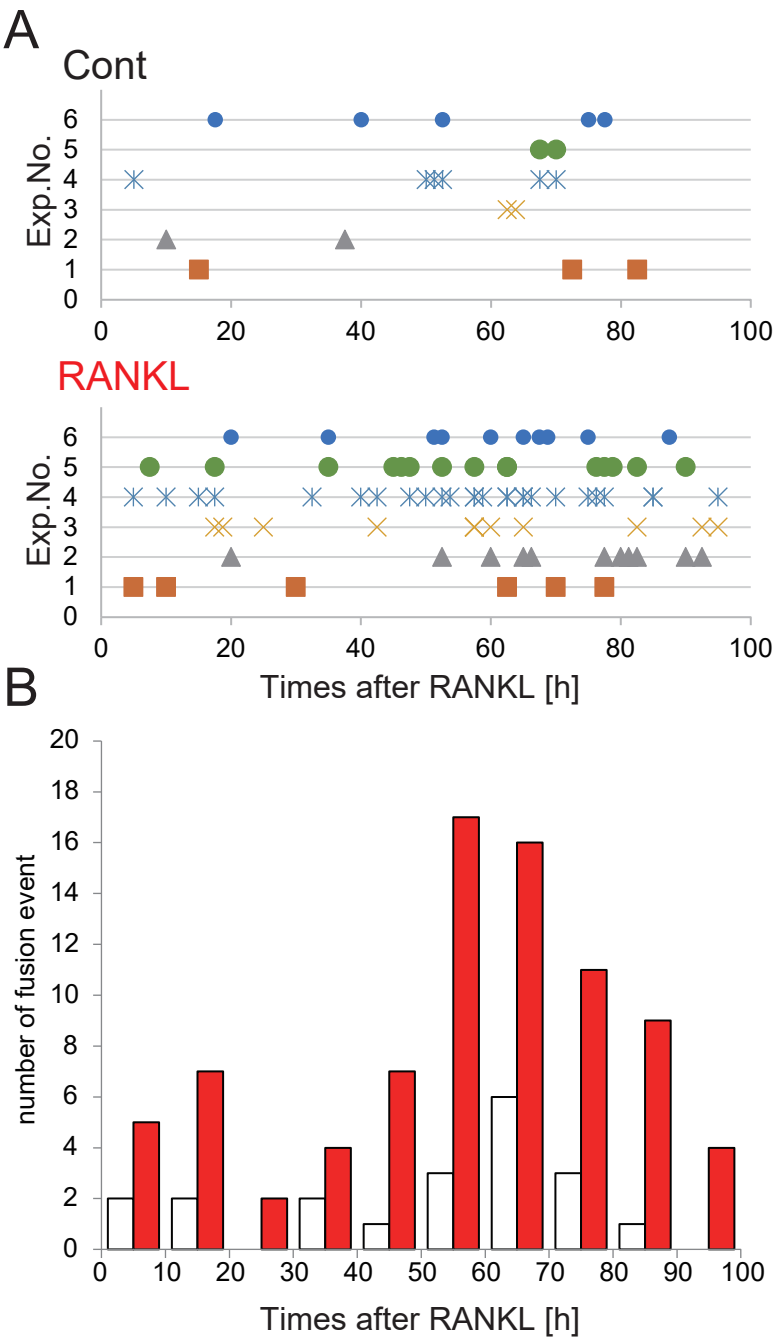

Fig. S4

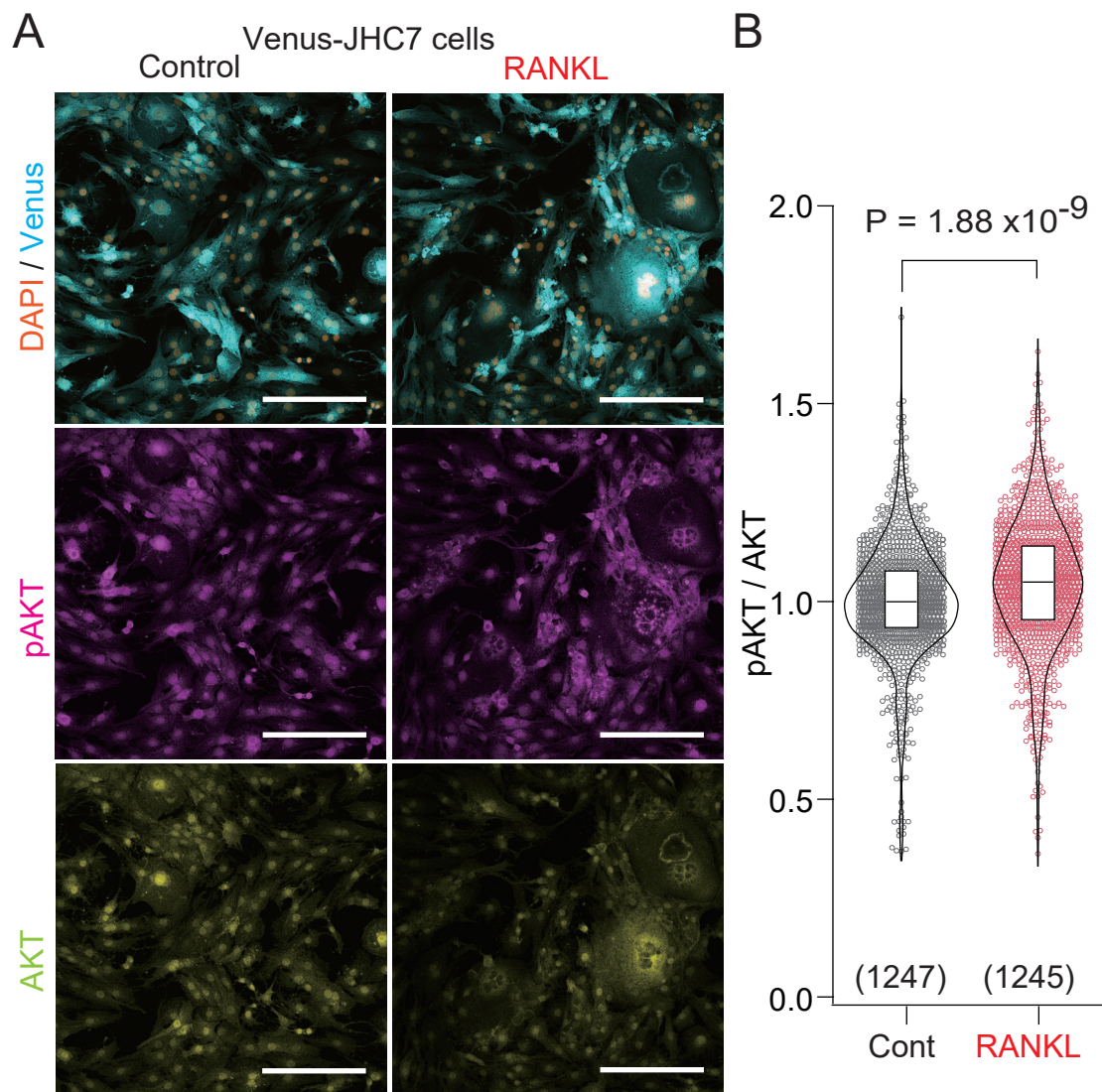

Fig. S5

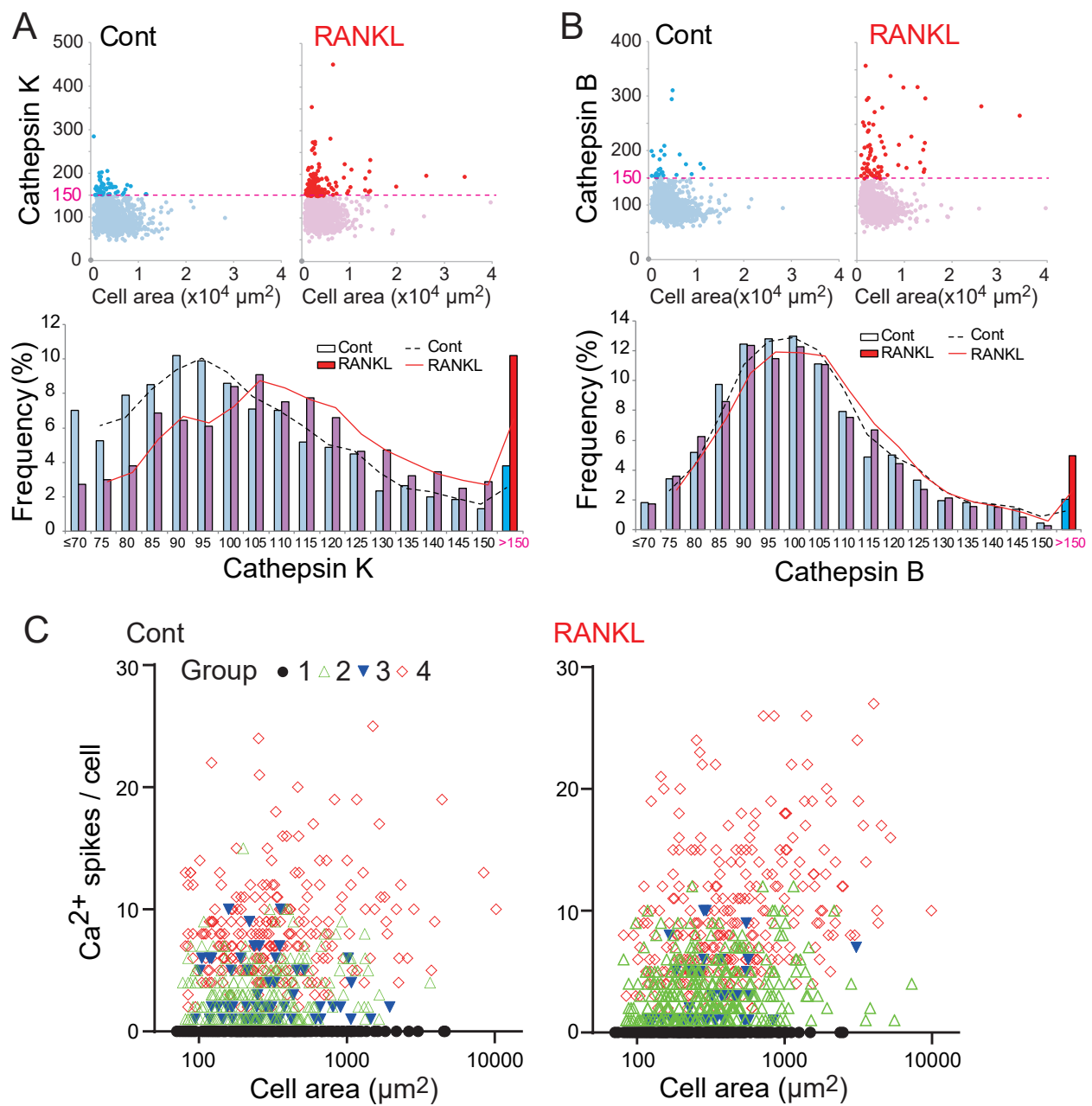

Fig. S6

A

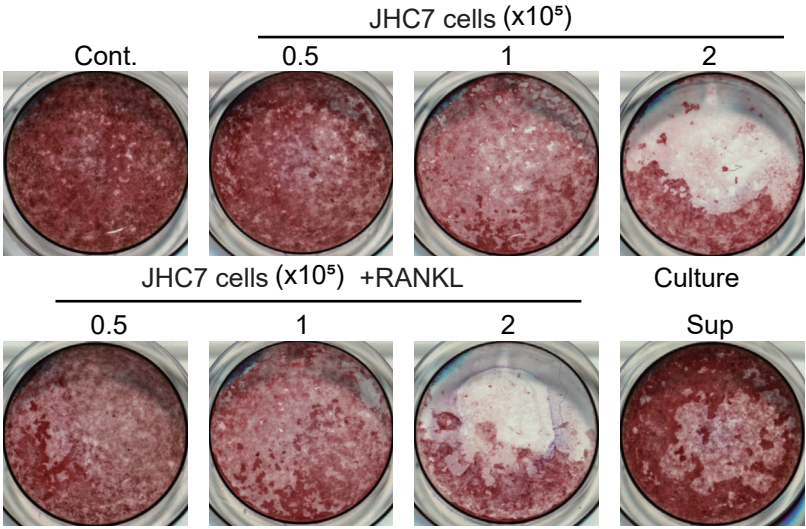

B

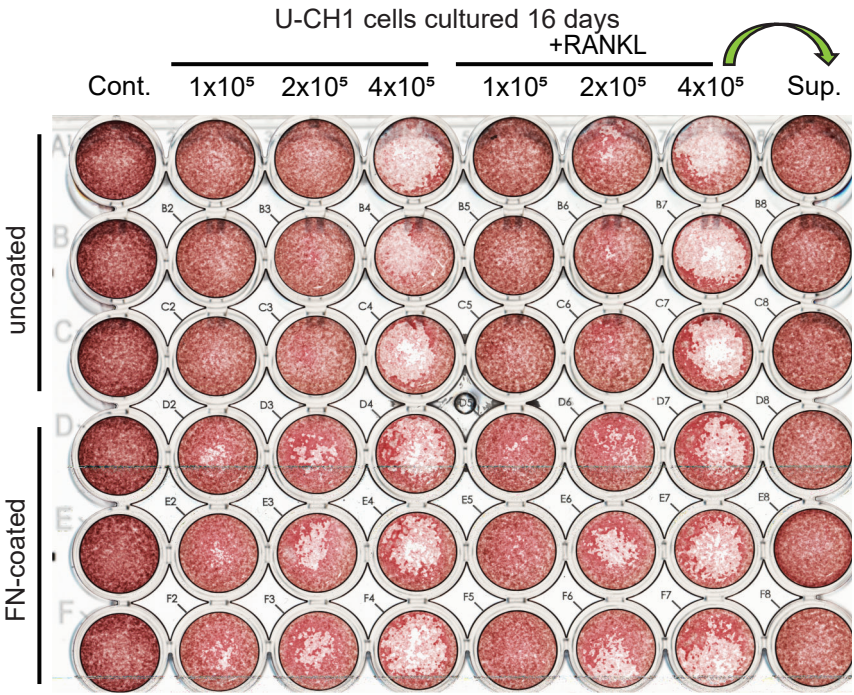

C

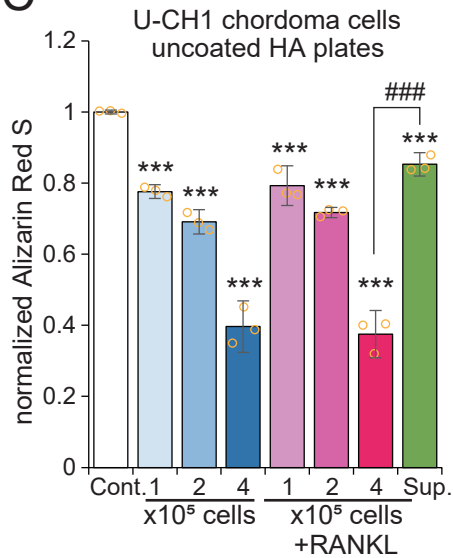

D

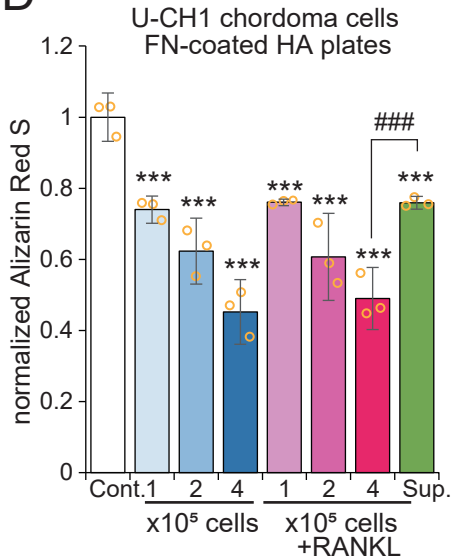

Supplement: Supplementary file 2 — Supplementary Material 2: Fig. S1 Brachyury-positive or -negative cells expressing TRAP at the tumor-bone boundary of chordoma tissues. (A) Immunostaining for control IgG (left) or brachyury (Brac; brown, right) in bone tissue section including chordoma cells. Scale bar, 100 µm. (B) Fluorescent immunostaining of chordoma tissue. Arrows indicate conventional osteoclasts expressing TRAP. Filled arrowheads indicate cells co-expressing brachyury and TRAP. Open arrowheads indicate conventional chordoma cells expressing brachyury. Dotted line, bone tissue. Magnified views of small white boxes in each panel are inserted. Scale bars in magnified views, 20 µm. (C) Quantitation of brachyury in TRAP-expressing cells in (A). Scatter plot of TRAP and brachyury expression in individual cells. TRAP-positive cells (5 chordoma specimens including bones) totalled 154. (D) Histogram showing distribution of brachyury-positive or -negative cells. Fig. S2 Brachyury expression in JHC7 cells. (A) Co-immunostaining of JHC7 cells using normal IgG. (B) Co-immunostaining of JHC7 cells using two anti-Brachyury antibodies, which recognize the different epitopes (AF2085: 2-202 aa, 1H9A2: 257–309 aa). Arrows, multinucleated large JHC7 cells stained with both brachyury antibodies in cytoplasm. Scale bar, 30 µm. Fig. S3 Time course of fusion events in JHC7 cell culture. (A) Each fusion event was plotted in chronological order after RANKL addition. (B) Histogram showing distribution of total fusion events every 10 hours in (A). Fig. S4 AKT phosphorylation in JHC7 cells. (A) Immunocytochemical analysis of levels of phospho- (red) and total (green) AKT in Venus-JHC7 cells, in the presence or absence of RANKL. Scale bar, 200 µm. (B) Quantitation of AKT phosphorylation in (A). Violin plots show distribution of AKT phosphorylation (phosphorylated AKT/total AKT). Data were normalized to the average ratio (phospho-AKT(Ser473P)/total AKT) seen in control cells. Total numbers of cells are indicated at the bot [file 13402_2024_946_MOESM2_ESM.pdf]
